# Navigating Ventricular Fibrillation in the Setting of Hypertrophy Cardiomyopathy and Commotio Cordis

**DOI:** 10.7759/cureus.79419

**Published:** 2025-02-21

**Authors:** Alena Gonzalez, Emmanuel Ukenenye, Selin Unal, Joseph Jayaraj

**Affiliations:** 1 Internal Medicine, Elmhurst Hospital, Queens, USA; 2 Internal Medicine, Brookdale University Hospital Medical Center, Brooklyn, USA; 3 Internal Medicine, State University of New York Downstate Health Sciences University, Brooklyn, USA; 4 Cardiology, State University of New York Downstate Health Sciences University, Brooklyn, USA

**Keywords:** commotio cordis, heart, hypertrophic cardiomyopathy, transthoracic echocardiography (tte), ventricular fibrillation

## Abstract

Ventricular fibrillation (VF) is a life-threatening cardiac arrhythmia characterized by asynchronous and disorganized contractions of the ventricular myocardium, leading to circulatory collapse, and if untreated, death. We present a case of a 30-year-old male who presented with VF following a soccer ball impact, revealing concurrent hypertrophic cardiomyopathy (HCM) and commotio cordis (CC). While both conditions have different mechanisms of action behind VF, management strategies for both encompass lifestyle adjustments, pharmacotherapy, and potential septal reduction procedures. Preventive measures such as safety protocols, implantable cardioverter-defibrillator (ICD) placement, and cardiopulmonary resuscitation (CPR) education are crucial. Overall, managing VF amid the complex interplay of HCM and CC emphasizes the need for tailored, multidisciplinary strategies to optimize patient outcomes.

## Introduction

Ventricular fibrillation (VF) is the most common arrhythmia observed in cases of circulatory collapse, accounting for approximately 30% of such incidents [1]. The underlying electrophysiological mechanisms include ectopic automaticity, reentry, and triggered activity, which can be exacerbated by myocardial hypertrophy, heart failure, and genetic predispositions. Among young athletes, hypertrophic cardiomyopathy (HCM) is the most common cause of SCD, while commotio cordis (CC) is the second most frequent cause.

VF is primarily influenced by the electrical heterogeneity and anisotropy of the ventricular myocardium, which can lead to irregular electrical conduction. For example, myocardial hypertrophy and heart failure can prolong action potential duration, contributing to the development of early after-depolarizations and increasing proarrhythmic susceptibility [1]. This is mainly due to electrical remodeling of the heart through activation of adenosine triphosphate (ATP)-sensitive potassium channels, which compromise repolarization reserve [1]. Repolarization reserve plays a significant role in VF genesis; it is defined as a defensive mechanism of the myocardium against arrhythmias, which can be compromised by congenital alterations in ion currents, heart failure, cardiac hypertrophy, bradycardia, and various pharmacological agents resulting in severely decreased in repolarization reserve, predisposing individuals to proarrhythmic [1-2]. Treatment of VF is according to Advanced Cardiovascular Life Support (ACLS) protocol, which consists of high-quality cardiopulmonary resuscitation (CPR) and early defibrillation, consistent with 150-360 J, followed by an investigation and study to determine the underlying cause of VF.

HCM and CC are both well-established causes of VF, posing significant challenges in clinical diagnosis and management when they coexist. We present the case of a 30-year-old male brought in by emergency medical services (EMS) for VF after being struck by a soccer ball in the chest, requiring defibrillation and intubation. This case illustrates the diagnostic and management challenges associated with VF when HCM and CC coexist.

## Case presentation

A 30-year-old male with a history of an unspecified childhood murmur collapsed after being struck in the chest by a soccer ball while playing soccer. Witnesses reported an immediate loss of consciousness. EMS arrived within five minutes, finding the patient unresponsive and in VF. Bystander CPR had not been performed prior to EMS arrival. He was defibrillated once, achieving a return of spontaneous circulation (ROSC) within 10 minutes of collapse. The patient was then intubated and transported to the emergency department (ED), where therapeutic hypothermia was initiated for neuroprotection following cardiac arrest.

**Figure 1:**
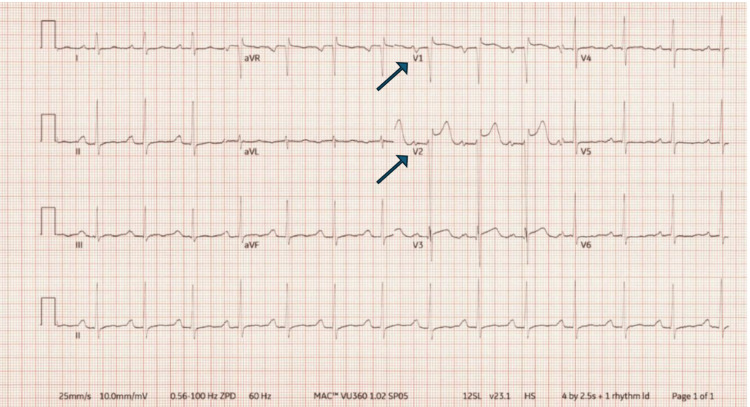
EKG showing sinus rhythm with first-degree AV block, ST elevation in V1 and V2, QTc of 480 ms.

Upon arrival, the patient exhibited jerky limb movements concerning new-onset seizures, leading to the administration of levetiracetam, propofol, and midazolam for sedation. Physical examination revealed a 3/6 systolic murmur at the lower left sternal border. Laboratory findings showed serum creatinine of 1.73 mg/dL, pH of 7.31, CO₂ of 52 mmHg, lactate of 1.5 mmol/L, and troponin of 0.036 ng/mL. Electrocardiography (ECG) showed sinus rhythm with first-degree atrioventricular (AV) block, ST elevation in V1 and V2, and a QTc of 480 ms (Figure 1). A chest X-ray revealed no intrapulmonary pathology.

**Figure 2:**
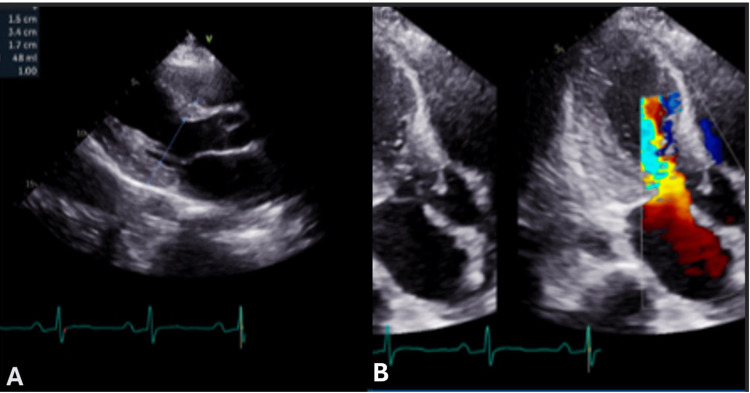
Parasternal long axis showing a septum and LV measurement of 1.5 cm consisting of LVH (A). Apical long axis showing an EF of 60%, diastolic dysfunction with increased mean LA pressure, and systolic anterior motion with LVOTO with a peak gradient of 111 mmHg (B). LA, left atrium; LV, left ventricle; LVOTO, left ventricular outflow tract obstruction

Neurology was consulted for neuro-prognostication and management of possible seizure disorder. They recommended continuing levetiracetam, and a CT scan of the head showed no acute signs of anoxic brain injury. Cardiology was consulted for a high concern of HCM and recommended cardio-familial genetic testing, transthoracic echocardiogram (TTE), and starting the patient on amiodarone to avoid arrhythmias. The patient was weaned off sedation and successfully extubated, with an unremarkable exam and no neurological deficits. TTE revealed severe left ventricular hypertrophy, ejection fraction of 60%, diastolic dysfunction with increased mean LA pressure, systolic anterior motion with left ventricular outflow tract obstruction (LVOTO) with a peak gradient of 111 mmHg (Figures 2A-2B).

Amiodarone was initiated acutely to suppress ventricular arrhythmias, given HCM’s known risk of malignant ventricular tachyarrhythmias, particularly post-cardiac arrest. Following extubation, neurological recovery was closely monitored using serial Glasgow Coma Scale (GCS) assessments and cognitive testing, which demonstrated intact memory, attention, and motor function. The patient was transitioned to metoprolol for rate control and to mitigate adrenergic triggers of arrhythmia. Given evidence of LVOTO on stress echocardiography, disopyramide was later added for its negative inotropic effects, helping to reduce dynamic obstruction and arrhythmia burden. The patient was transferred to the cardiac intensive care unit for planned dual-chamber implantable cardioverter-defibrillator (ICD) implantation, which was performed without complications. He was discharged home with instructions for outpatient cardiology and genetic follow-up.

## Discussion

Pathophysiology and arrhythmogenesis of HCM

HCM is a common cardiac disorder and a significant cause of sudden cardiac death, affecting both men and women across all races and physical fitness levels, including well-trained athletes. It typically results from asymmetric septal hypertrophy, obstructing blood flow through the left ventricular outflow tract [3]. HCM may result from genetic mutations in genes encoding sarcomere proteins, leading to structural abnormalities in myofibrils and myocytes, often inherited in an autosomal dominant manner. Other causes include heightened sympathetic stimulation and coronary artery disease.

Pathophysiologically, HCM can be classified into obstructive or non-obstructive, with dynamic outflow obstruction caused by the systolic anterior motion of the anterior leaflet of the mitral valve. This obstruction is dependent on contractility and loading conditions and may lead to ventricular arrhythmias and sudden death due to increased myocardial ischemia. Researchers investigated intra-ventricular conduction abnormalities while performing an invasive electrophysiological study to understand their role in ventricular arrhythmias in HCM. They studied 37 HCM patients, including those with VF, ventricular tachycardia (VT), or a family history of sudden death. Telemetry was recorded during pacing in the right ventricle, revealing increased dispersion and inhomogeneity of intraventricular conduction in patients at risk of sudden death [4]. These findings suggest that conduction abnormalities may contribute to arrhythmogenesis in HCM.

An example of conduction abnormalities that lead to ventricular arrhythmia is delayed after depolarization. Delayed after-depolarization is a phenomenon in which a cardiac myocyte's depolarization occurs after an action potential is completed. Thus, delayed after-depolarization leads to ventricular tachyarrhythmias and sudden cardiac death.

Apart from HCM, one of the known conditions that includes delayed after-depolarization as part of its pathophysiology is catecholaminergic polymorphic VT (CPVT). CPVT is a rare genetic disorder causing exercise- or stress-induced VT due to abnormal calcium signaling, requiring early diagnosis and management with beta-blockers (BBs), calcium channel blockers, or ICDs to prevent sudden cardiac death [5].

Management strategies of HCM

Management for HCM consists of a combination of lifestyle modifications, medications, and, occasionally, invasive procedures [6]. These multidisciplinary treatment modalities aim to ameliorate symptoms, suppress disease progression, and improve long-term outcomes in patients with HCM, especially when LVOTO is present. Lifestyle modifications play a vital role in wholesome management; strenuous physical activity is to be avoided due to the increased risk of arrhythmias and sudden cardiac arrest (SCA). Frequent monitoring of symptoms and cardiac activity enables the early identification of disease advancement and the optimization of treatment approaches.

Genetic counseling and family member screening enable the identification of individuals at risk of inheriting HCM, facilitating early risk stratification and intervention [6-7]. BBs represent a cornerstone in the pharmacological management of HCM. They exert their therapeutic effect by reducing myocardial contractility and chronotropy. This dual action acts to alleviate symptoms associated with HCM, such as chest pain, dyspnea, and palpitations. In addition, BB blunts the excessive myocardial contractility during systole, which improves the dynamic LVOTO [7]. Verapamil and diltiazem (calcium channel blockers) provide another pharmacotherapeutic avenue for management. These medications induce vasodilation and reduce myocardial contractility. As a result, they mitigate LVOTO and result in improvement in cardiac relaxation. This leads to improvement in symptoms and enhancement of exercise tolerance [7-8].

On the other hand, class I anti-arrhythmic medication, disopyramide, may be utilized for symptomatic treatment and hemodynamic impairment in patients with HCM. Disopyramide reduces myocardial contractility and heart rate via inhibition of myocardial sodium channels, thereby ameliorating LVOTO and enhancing diastolic filling. This therapy represents an adjunctive strategy for managing symptomatic patients [8]. Septal reduction therapies, such as alcohol septal ablation and septal myectomy, are recommended in severely symptomatic patients who failed pharmacotherapy, providing a more definitive treatment option [7-8]. Mavacamten is a promising drug for treating HCM. The EXPLORER-HCM trial investigated the drug's safety and efficacy in 251 patients with symptomatic obstructive HCM. Mavacamten, a cardiac myosin inhibitor approved by the FDA in 2022, has demonstrated efficacy in reducing LVOTO in HCM. However, it requires monitoring for systolic dysfunction and has insurance coverage limitations. The drug inhibits the activity of the cardiac myosin protein, which reduces hypercontractility of the heart muscles. Further studies are required to confirm the drug's long-term safety and efficacy [9]. 

Finally, placement of ICD is recommended for patients predisposed to ventricular arrhythmias. The cardiac rhythm is continuously monitored by this device, which delivers electrical signals to abort fatal arrhythmias, thereby avoiding sudden cardiac arrest [7].

Pathophysiology and arrhythmogenesis of CC

On the other hand, CC is a fatal mechano-electric syndrome reported as the second most common cause of sudden cardiac death in young athletes without structural heart disease, HCM being the number one [3-10]. Over 190 cases have been reported in the United States, with 47% occurring during athletic activities, making it the second most common cause of sudden cardiac death in athletes. Defibrillation is effective in only 15% of cases, with a 25% survival rate when administered within three minutes. The overall survival rate remains relatively low, as many cases go unwitnessed or experience delayed defibrillation [10]. 

The immediate trigger for VF in CC is likely a focal phenomenon induced by direct impact, like a premature ventricular contraction seen in electrophysiology labs or cardiac catheterization procedures [11]. Alternatively, it could be an afterdepolarization induced by changes in current flow, similar to the R-on-T phenomenon observed in acute ischemia or in long-QT syndrome. Internally, VF may be more organized, with the presence of a stationary or roaming mother rotor or multiple random wandering wavelets. VF in CC may appear like other conditions like acute ischemia, electric shock, long-QT syndrome, or Brugada syndrome [11-12]. It is also hypothesized that VF in CC may result from acquired abnormalities in repolarization caused by impact, leading to increased heterogeneity of repolarization and making the myocardial substrate prone to reentrant arrhythmias [11]. However, temporal alteration of the myocardial substrate alone is not sufficient; there must also be a trigger, such as an afterdepolarization or premature ventricular depolarization induced by the impact. Ion channels involved in altered repolarization, particularly those affected by stretch or pressure changes, are potential candidates for the mechanism of VF in CC [11]. 

Management strategies of CC

Survival in CC is strongly dependent on rapid defibrillation and effective CPR. The use of an automated external defibrillator (AED) within the first few minutes of collapse significantly improves survival outcomes as well [13]. Initiatives like educating coaches, athletic personnel, teachers, parents, and students in CPR techniques are paramount to equip them with the necessary skills to respond effectively in emergency situations [14]. Adaptation of athletic activities and adoption of safe playing techniques are crucial preventive measures to mitigate the risk of chest trauma. Moreover, the utilization of safety baseballs or t-balls, particularly for younger children, represents an additional preventive measure. These balls, being softer and more flexible than standard baseballs, reduce the likelihood of causing significant chest trauma, thereby contributing to injury prevention during athletic activities [15].

In CC survivors with an idiopathic cardiac etiology, the routine use of ICD for primary transient does not necessarily indicate an underlying arrhythmogenic substrate [15-16]. As a result, ICD implantation in such cases may not offer significant benefit, rather it may expose patients to unnecessary risks that accompany device implantation and management. While ICDs are not routinely implanted in CC with unknown cardiac cause, a case-by-case basis in patients with documented underlying cardiac abnormalities that increase the risk of SCA may be utilized [15-16]. The decision for ICD implantation in such cases should be made meticulously, balancing the potential benefits against the risks and considering the individual patient's clinical profile and preferences.

## Conclusions

Managing VF in the setting of both HCM and CC requires a multidisciplinary approach. While acute management prioritizes defibrillation and neuroprotection, long-term strategies must address arrhythmic risks through tailored pharmacotherapy, lifestyle modifications, and ICD placement when indicated. The role of emerging therapies like Mavacamten is promising, but ongoing surveillance is necessary. Additionally, improving sports safety measures and CPR training remains critical in preventing CC-related deaths. This case underscores the importance of individualized care strategies to optimize patient outcomes.
